# Impact of the COVID-19 pandemic on university students’ psychological distress, well-being, and utilization of mental health services in the United States: populations at greatest risk

**DOI:** 10.3389/fpubh.2024.1442773

**Published:** 2024-10-30

**Authors:** Elaine Cooper Russell, Tolulope M. Abidogun, Lisa L. Lindley, Kenneth W. Griffin

**Affiliations:** ^1^Department of Global and Community Health, George Mason University, Fairfax, VA, United States; ^2^Department of Community and Population Health, Lehigh University, Bethlehem, PA, United States

**Keywords:** mental health, mental health service utilization, United States university/college students, psychological distress and well-being, COVID-19 pandemic

## Abstract

**Introduction:**

The COVID-19 pandemic led to major disruptions in the lives of university students, which is a population that is already at a greater risk of mental health concerns. Little is known about how the pandemic impacted distress and mental health services utilization among university students across the United States.

**Methods:**

Using survey data from the National College Health Assessment, both before the COVID-19 pandemic (pre-March 2020, *n* = 88,986) and during the pandemic (Spring 2021, *n* = 96,489), the present study examined mental health symptoms and utilization of mental health services among undergraduate students attending four-year universities in the United States.

**Results:**

There were notable increases in measures of psychological distress and reductions in well-being from before the pandemic to during the pandemic. However, overall utilization rates of mental health services slightly decreased from pre-pandemic to during the pandemic. Predictors of severe psychological distress included those who experienced loneliness, COVID-19 related stressors, and loss of a loved one from COVID-19. COVID-related stressors and loneliness were associated with higher utilization rates of mental health services, while well-being and resilience were associated with lower utilization rates.

**Discussion:**

Analyses revealed that several demographic groups were at an elevated risk for severe psychological distress, including non-binary, female, and sexual minority students, and especially those who identify as both non-binary and non-heterosexual. Results indicated that students of color, especially female students of color, were less likely to receive mental health services. Future research is needed to increase our understanding of the barriers to mental health service use among high-risk university students.

## 1 Introduction

### 1.1 Mental health in United States college students

The mental health crisis among United States college students is a critical public health concern. College students often experience a variety of stressors including increased academic demands, homesickness, social pressures, financial stress, and other factors that can increase the risk for mental health problems during the transition to young adulthood. Over the past 2 decades, rates of depression, anxiety, substance abuse, and suicidal ideation have reached record-high levels in United States college students (1). The COVID-19 pandemic introduced a host of new challenges that exacerbated the mental health crisis among college students, and several indicators of poor psychological adjustment worsened during this time (2). The COVID-19 pandemic brought on much uncertainty, loneliness, health impacts, and financial loss, which in turn led to spikes in depression, anxiety, post-traumatic stress disorder, and suicidal ideation (3). In 2021, more than 60% of college students met the criteria for at least one mental health concern, and three-quarters of students reported periodic moderate to severe psychological distress (4). Colleges saw a 66% increase in depression and higher stress levels during the pandemic compared to pre-pandemic times (5). If left untreated, the consequences of poor mental health can extend into adulthood—thus impairing physical health as well, and limit ones’ opportunities toward a fulfilling life (6).

Some minoritized groups were disproportionally impacted by mental health concerns during the COVID-19 pandemic. Sexual and gender minorities displayed more frequent symptoms of depression and anxiety than their heterosexual and cisgender counterparts (7), and one study found the lowest mental health and highest academic stress in non-binary students (8). Additionally, when assessing past-year diagnosis and treatment of depression, anxiety, and suicidal ideation, Samek et al. (9) found significantly greater odds of each of these mental health indicators in non-heterosexual and non-binary students. The COVID-19 pandemic also had more pronounced negative effects on female university students, even when controlling for distress prior to the pandemic, with factors such as academic stress and social isolation being major contributors (8, 10).

Further, increased psychological distress has been reported among freshman students and those closer to graduating, low-income students, and those with family members who experienced adverse health outcomes due to COVID-19 (8, 11–13). On the other hand, frequent in-person social interactions was found to be protective against psychological distress, and was associated with lower levels of depressive symptoms in college students (14). Prior studies have also found that higher parental education, especially of the mother, is associated with better mental well-being in college students (15, 16). With access to quality mental health treatment being a challenge in the United States, parental education could be an important protective factor in helping better connect adolescents to treatment (17).

Intersectionality and minority stress theory could be used to explain these mental health impacts on disproportionally affected populations. Intersectionality is a theory used to explain the unique social, structural, and individual experiences (particularly related to stigma and discrimination) among individuals with multiple identities and social classifications (18). Additionally, minority stress theory explains how chronic exposure to social stress due to stigma and discrimination may lead to poor health outcomes among minorities, including sexual and gender minority populations (19). This study will examine how intersections between race, gender, and sexual minority status are associated with psychological distress and well-being. Psychological distress is a state of mental and emotional suffering, to include symptoms of feeling depressed and helpless, and in some cases may indicate the onset of major depressive and/or anxiety disorder (20). Conversely, well-being encompasses positive mood states, such as happiness, a sense of purpose and meaning, and contentment, along with low levels of distress (20).

### 1.2 Mental health service utilization

The pandemic intensified barriers to receiving mental health treatment for college students, as in-person congregations were prohibited and/or strongly discouraged. For those who did seek treatment, the increased burden of mental distress during the COVID-19 pandemic led to campus resources being stretched thin. Greater demands for campus resources can be challenging to meet with limited university funding available, causing long wait times and frustration to students, which may discourage them from seeking help (21). This can further exacerbate barriers for students suffering the most, as research indicates that young adults who report more psychological distress are less likely to seek help (22). Stigma is another major barrier to seeking help for mental health struggles, especially among ethnic minority students (8, 23).

Despite worsening mental health symptoms within young adults in the United States, of those aged 18–25, less than half (44.6%) of those with a mental illness received treatment in the past year (24). Prior to the pandemic, research indicated that less than a quarter of university students received the necessary treatment for their mental health (22). One study conducted during the early stages of the pandemic found that 60% of students with moderate to severe stress, anxiety, or depression have never utilized on-campus mental health services, and more than two-thirds of students never used off-campus mental health services (13).

The present study is the first to utilize a national dataset to examine changes in United States university students’ psychological well-being and utilization of mental health services from pre-pandemic to during the pandemic. Additionally, this study examines pertinent demographic groups and psychosocial factors that predict severe psychological distress and mental health services utilization at the peak of the pandemic. Using nationally representative data of undergraduate college students attending 4-year universities, we aimed to (1) examine mental health symptoms and utilization of mental health services among undergraduate students; and (2) identify demographic and psychosocial factors that predict severe psychological distress and mental health services utilization during the pandemic. This research study is important in helping to identify students who may be at the greatest risk of psychological distress and least likely to access necessary mental health services. This study also examines how various demographic variables and social identities, including gender, race/ethnicity, and sexual orientation, may be associated with one’s mental health and treatment.

## 2 Materials and methods

### 2.1 Data source and sample

Data used in this study were obtained from the American College Health Association National College Health Assessment (ACHA-NCHA III). ACHA-NCHA is a bi-annual cross-sectional survey administered by post-secondary institutions to college students across the United States on key topics including mental health, substance use, and sexual health. The ACHA-NCHA questionnaire has been revised since it was initiated in 2000, with the most recent version—ACHA-NCHA III—deployed in Fall 2019. Due to the COVID-19 pandemic, data collection for Spring 2020 was concluded before March 16, 2020, i.e., before the outbreak of the Sars-CoV-2 virus in the United States. For this study, we utilized data for Fall 2019 (*n* = 53 4-year universities), Spring 2020 (*n* = 71 4-year universities), and Spring 2021 (*n* = 130 4-year universities). The sample sizes for Fall 2019 (*n* = 38,679) and Spring 2020 (*n* = 50,307) were small compared to that of Spring 2021 (*n* = 96,489). Thus, we combined Fall 2019 and Spring 2020 data to represent the “pre-COVID-19” timepoint and Spring 2021 data was used to represent the “peak COVID-19” timepoint. Data for Fall 2020 were not included in our sample because the sample size was small due to pandemic disruptions (*n* = 13,373), and thus possibly unrepresentative. Inclusion criteria for this study included full-time undergraduate students between the ages of 18–24, who were enrolled in 4-year (or more) United States institutions.

First, we analyzed data on mental health status and mental health care utilization for the pre-COVID-19 and peak COVID-19 timepoints to measure trends in mental health status and mental health services utilization before and after the onset of the pandemic. Next, we focused on the Spring 2021 sample to examine predictors of mental health status and utilization of mental health services.

For the analyses focused on the pre- and peak-COVID timepoints, the study sample included 58,137 respondents in the pre-COVID-19 timepoint and 57,281 respondents in the peak COVID-19 timepoint after filtering for the inclusion criteria. For the latter time point (Spring 2021), the sample was primarily female (68.1%), about half were White (53.6%), and nearly three quarters (74%) of the sample identified as heterosexual/straight. For year in school, the sample contained a fairly even representation of academic class levels, to include 27.2% freshman, 23% second year students, 26.1% third year students, and 19.5% seniors; 4.2% of students were in their 5^th^ or greater year of their undergraduate degree. For race/ ethnicity, 53.6% of the sample was White, and 46.4% were non-White. Since all data were de-identified, this analysis was exempt from human subject review.

### 2.2 Demographic variables

#### 2.2.1 Race/ethnicity

Students reported their race or ethnicity as White (53.6%), Asian (15.7%), Hispanic (15.2%), Black (3.3%), Multi/Biracial (10.6%), and Other (1.7%—included American Indian or Native Alaskan, Middle Eastern or Arab Origin, Native Hawaiian or Other Pacific Islander, and not listed). For linear regression models, we recoded race/ethnicity as a dichotomous variable indicating White or non-White.

#### 2.2.2 Gender identity

Students reported their gender as male (27.4%), female (68.1%), or non-binary (4.5%).

#### 2.2.3 Sexual orientation

Students were asked to describe their sexual orientation. For analysis purposes, we dichotomized sexual orientation into heterosexual (74%) or non-heterosexual (26%).

#### 2.2.4 Parental education

Students were asked about the highest level of education completed by either of their parents or guardians. We created a dichotomous variable indicating whether students reported having at least one parent with a bachelor’s degree or higher (61.3%).

### 2.3 Measures of mental health status

#### 2.3.1 Psychological distress

The six-item Kessler Psychological Distress Scale (K6) is a validated instrument used to assess risk for nonspecific serious mental illness over the past month. Respondents were asked to respond to the following questions: “During the past 30 days, about how often did you feel (1) nervous, (2) hopeless, (3) restless or fidgety, (4) so depressed that nothing could cheer you up, (5) that everything was an effort, and (6) worthless.” Response options were on a five-point Likert scale from “All of the time” (4) to “None of the time” (0), with scores for each item ranging from 0 to 4. Individual scores were added together to yield total scores ranging from 0 to 24, with higher scores indicating worse psychological distress, and a score of 13+ designates severe psychological distress. For logistic regression analyses, severe psychological distress was used as a primary response variable in analyses examining psychological distress. This scale has a Cronbach’s alpha of 0.89.

#### 2.3.2 Loneliness

The Short UCLA Loneliness Scale (ULS3) is a validated instrument used to objectively measure social isolation in large-scale surveys. Respondents were asked to respond to the following questions: “How often do you feel that you lack companionship? How often do you feel left out? How often do you feel isolated from others?” Responses included “Hardly ever” (1), “Some of the time” (2), and “Often” (3). ULS3 scores range from 3 to 9, with higher scores reflecting higher levels of loneliness. Scores between 3 and 5 indicate a negative screen for loneliness, and those ranging from 6 to 9 indicate a positive screening for loneliness. This scale has a Cronbach’s alpha of 0.81.

#### 2.3.3 Overall stress

The study included a one-item measure of overall stress. Respondents were asked to respond to the following question: “Within the last 30 days, how would you rate the overall level of stress you have experienced.” Responses included “No stress” (1), “Low stress” (2), “Moderate stress” (3), and “High stress” (4). For reporting purposes, we dichotomized this variable into none to low stress, and moderate to high stress.

#### 2.3.4 Psychological well-being

The eight-item Diener Psychological Well-Being (Diener PWB) Scale is a validated self-report instrument to measure well-being in key aspects of life, to include relationships, self-esteem, purpose, and optimism. Respondents were asked to indicate how well they agreed with eight questions, including: “I lead a purposeful and meaningful life,” “My social relationships are supportive and rewarding,” and “I am engaged and interested in my daily activities.” Each item was measured using a seven-point Likert scale ranging from “strong disagreement” (1) to “strong agreement” (7). The Diener PWB generates a score between 8 and 56, with higher scores reflecting a higher level of psychological well-being. This scale has a Cronbach’s alpha of 0.93.

#### 2.3.5 Resilience

The Connor-Davidson Resilience Scale (CD-RISC2) is a short form of the original Connor-Davidson Resilience Scale used to assess resilience (the ability to cope with stress) and adaptability. The CD-RISC2 is a validated instrument with a high internal reliability that assesses resilience using two questions: (1) “I am able to adapt when changes occur,” and (2) “I tend to bounce back after illness, injury, or other hardships.” Responses options are on a five-point Likert scale ranging from “Not at all true” (0) to “True nearly all the time” (4). The two items were summed to create a single overall score ranging between zero and eight, with higher scores reflecting greater resilience. In the ACHA-NCHA instrument, resilience and adaptability were assessed over the past month. This scale has a Cronbach’s alpha of 0.78.

### 2.4 COVID-19 pandemic

#### 2.4.1 COVID-19 stressors

Students were asked how much they were concerned with the following over the past 30 days: (1) how long the COVID-19 pandemic will last, (2) that they will get COVID-19, (3) that they will get COVID-19 again, (4) someone they care about will get COVID-19, (5) someone they care about will die from COVID-19, (6) not being able to spend time with the people they care about, and (7) uncertainty of the future. Response options were on a Likert scale ranging from “not concerned at all” (1) to “extremely concerned” (5). This scale has a Cronbach’s alpha of 0.83.

#### 2.4.2 Lost a loved one from COVID-19

Students were asked whether they had a loved one, close family member, or friend die due to COVID-19. Respondents were asked to indicate “Yes” or “No.”

### 2.5 Measures of mental health services utilization

#### 2.5.1 Utilization of mental health services

Students were asked, “Within in the last 12 months, have you received psychological or mental health services (in-person or via telehealth)?” The survey did not differentiate between in-person and telehealth services, and both are factored together in this analysis. Past year utilization of mental health services was used as the second primary response variable in logistic regression analyses. For students who reported yes to receiving mental health services in the past 12 months, they were asked to specify where these services took place. Options included: (1) My current campus health and/or counseling center, (2) A mental health provider in the local community near my campus, (3) A mental health provider in my hometown, and (4) A mental health provider not described above (“other”).

Additionally, students were asked to report whether they sought treatment in the past 12-month for a diagnosis of depression or anxiety. Students were asked if they have ever been diagnosed by a healthcare or mental health professional with ongoing or chronic depression or anxiety, and whether they had an appointment and/or discussion with a healthcare or mental health professional for these conditions within the last 12 months.

#### 2.5.2 Health insurance

Students were asked about their primary source of health insurance. Options included: (1) being on a university student health insurance plan, (2) covered by a parent/guardian, (3) covered by an employer (including a spouse’s employer-based plan), (4) Medicaid, Medicare, SCHIP, or VA/Tricare coverage, (5) does not have health insurance, etc. For the purposes of regression analyses, we created a dichotomous variable indicating whether or not students had health insurance.

## 3 Results

### 3.1 Pre-COVID-19 and peak COVID-19

**Figure 1:**
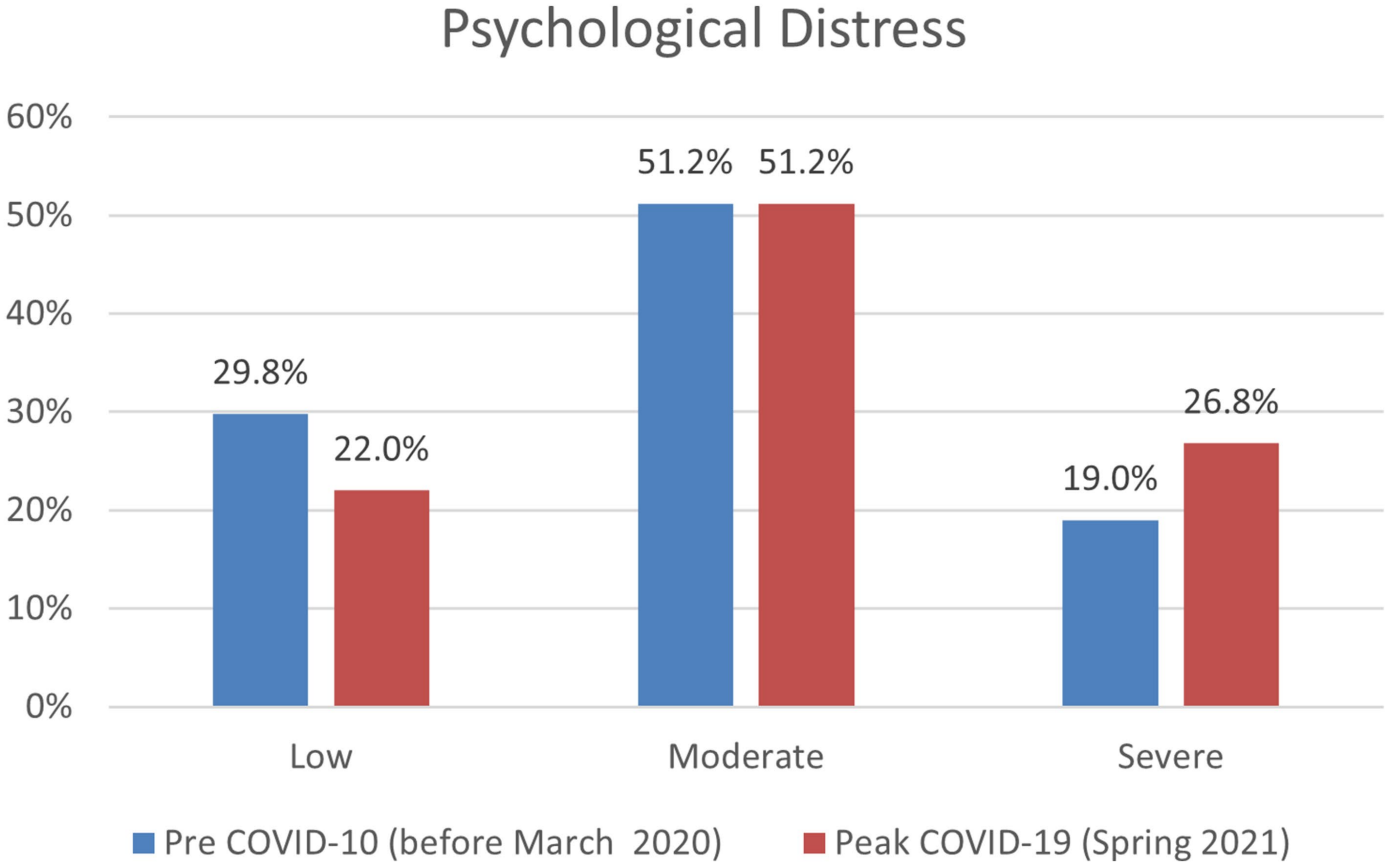
Psychological distress (K6 score) categorized by low, moderate, and severe for pre and peak COVID-19.

As displayed in Table 1, we conducted a series of independent sample *t*-tests to analyze if mental health status and utilization of mental health services differed across the pre-pandemic and peak COVID-19 timepoints. There was a statistically significant increase in rates of psychological distress from pre (M = 7.98, SD = 5.20) to peak (M = 9.26, SD = 5.49) COVID-19, indicating greater psychological distress. Additionally, when looking at rates of students suffering from severe psychological distress (a score of 13+ on the K6 scale), rates increased from 19.0% pre-pandemic to 26.8% in Spring 2021 (Figure 1). When analyzing other mental health indicators, statistically significant decreases were seen in resilience (M = 5.98, SD = 1.50 to M = 5.92, SD = 1.55) and psychological well-being (M = 46.03, SD = 8.18 to M = 43.83, SD = 8.70), while loneliness increased (M = 5.49, SD = 1.86 to M = 5.76, SD = 1.87). Additionally, during the peak of COVID-19, 82.4% of students reported experiencing moderate to high stress over the past 30 days, compared to 75.8% pre-COVID-19, which was also found to be statistically significant.

**Table 1:** Mental health status and utilization of mental health services, pre-pandemic and peak-COVID-19.

	Pre-pandemic: Fall 2019 and Spring 2020 (*n* = 58,137)	Peak COVID-19: Spring 2021 (*n* = 57,281)	Statistical significance
*Indicators of poor mental health status*			
Psychological distress (Kessler 6)	M = 7.98, (SD = 5.20)	M = 9.26, (SD = 5.49)	*t* (113548.50) = −40.42, *p* < 0.001
Loneliness (UCLA)	M = 5.49, (SD = 1.86)	M = 5.76, (SD = 1.87)	*t* (115041.34) = −24.08, *p* < 0.001
Moderate to severe stress	75.8%	82.4%	*X*^2^(1) = 751.70, *p* < 0.001
*Indicators of positive mental health status*			
Well-being (Diener)	M = 46.03, (SD = 8.18)	M = 43.83, (SD = 8.70)	*t* (113891.71) = 44.18, *p* < 0.001
Resilience	M = 5.98, (SD = 1.50)	M = 5.92, (SD = 1.55)	*t* (114,899) = 6.84, *p* < 0.001
*Utilization of mental health services*			
Utilized mental health (MH) services in the past 12-month	28.4%	27.8%	*X*^2^(1) = 5.77, *p* = 0.016
On campus	58.6%	44.4%	*X*^2^(1) = 626.68, *p* < 0.001
Local community near campus	24.8%	21.9%	*X*^2^(1) = 36.12, *p* < 0.001
Hometown	45.1%	49.3%	*X*^2^(1) = 54.33, *p* < 0.001
Other	21.5%	28.5%	*X*^2^(1) = 172.34, p < 0.001
Sought treatment in the past 12-month for diagnosed anxiety	18.9%, (SD = 0.39)	19.9%, (SD = 0.40)	*X*^2^(1) = 15.21, *p* < 0.001
Sought treatment in the past 12-month for diagnosed depression	15.1%, (SD = 0.36)	15.9%, (SD = 0.37)	*X*^2^(1) = 21.40, *p* < 0.001

Spring 2020 data were collected prior to March 16, 2020.

We ran a series of chi-square tests to compare utilization of mental health services from prior to the pandemic to during. Overall, university students accessed services slightly less during the peak of the pandemic (28.4–27.8%). Significant changes were found in where students accessed mental health services. Of those who received mental health services, rates of on-campus use of mental health services decreased from 58.6 to 44.4%, and rates of utilizing services in the local community near campus decreased from 24.8 to 21.9%. Conversely, students began using mental health services in their hometown more frequently, as rates increased from 45.1% prior to the pandemic to 49.3% during peak COVID-19. During the pandemic, students also sought more treatment for diagnosed anxiety (18.9–19.9%) and depression (15.1–15.9%).

#### 3.1.1 Peak pandemic timepoint

After assessing trends in psychological well-being and utilization of mental health services from pre-pandemic to peak pandemic timepoints, we focused the remaining analyses on Spring 2021 data to identify factors most associated with severe psychological distress and utilization of mental health services. In Spring 2021, more than a quarter (26.8%) of students reported severe psychological distress. Approximately one in every six students (15.8%) reported losing a loved one from COVID-19, and 27% of those who lost a loved one did seek mental health treatment in the past year. Nearly two thirds of students (61.3%) reported that at least one parent/guardian has a bachelor’s degree. For health insurance, many students were still covered under their parents’ health insurance (76.6%), had a plan through the university (7.9%), or had Medicare, Medicaid, SCHIP, or VA/Tricare coverage (8.4%); only 2.3% of students reported not having health insurance.

### 3.2 Mental health symptoms (severe K6)

As indicated in Table 2, one-way ANOVA tests were conducted for demographic variables and levels of psychological distress for Spring 2021 data. With higher K6 scores indicating worsened psychological distress, males had the lowest levels of psychological distress (M = 7.91, SD = 5.44), followed by females (M = 9.57, SD = 5.28), and non-binary students displayed the greatest levels of distress (M = 12.51, SD = 5.39); significant differences were found between and within all gender groups. Regarding sexual orientation, psychological distress was significantly higher for non-heterosexual students (M = 11.69, SD = 5.33) compared to heterosexual students (M = 8.40, SD = 5.3). When analyzing race/ethnicity data, students classified as “Other” had the highest psychological distress score (M = 10.60, SD = 6.13), followed by Hispanic students (M = 9.76, SD = 5.78). There was a statically significant difference (*p* < 0.001) in average K6 for non-White students (M = 9.71, SD = 5.64) compared to White (M = 8.85, SD = 5.32).

**Table 2:** Psychological distress (mean Kessler 6 score) by demographic subgroup.

During pandemic: Spring 2021				
Demographic	Sub-group	*N*	Mean (SD)	Statistical significance
Gender	Male	15,571	7.91 (5.44)	*F*(2, 56,274) = 1,010.17, *p* < 0.001
	Female	38,784	9.57 (5.28)	
	Non-binary	2,569	12.51 (5.39)	
Sexual orientation	Heterosexual	41,928	8.41 (5.30)	*F*(1, 56,447) = 4,101.77, *p* < 0.001
	Non-heterosexual	14,521	11.69 (5.33)	
Year in school	1st year	15,535	9.17 (5.54)	*F*(4, 56,624) = 17.48, *p* < 0.001
	2nd year	13,200	9.48 (5.47)	
	3rd year	14,969	9.34 (5.49)	
	4th year	11,182	8.95 (5.39)	
	5th year	2,395	9.53 (5.76)	
Race/ethnicity	White	29,869	8.85 (5.32)	*F*(5, 55,064) = 74.72, *p* < 0.001
	Asian	8,731	9.65 (5.48)	
	Black or African American	1,813	9.40 (5.86)	
	Hispanic	8,449	9.76 (5.78)	
	Biracial or Multiracial	5,894	9.69 (5.53)	
	Other	940	10.6 (6.13)	
	Non-White total	25,827	9.71 (5.64)	

We conducted a series of logistic regression analyses examining severe psychological distress that included the following predictors: year in school; parent bachelor’s degree; the psychological well-being, resilience, loneliness, and COVID-19 stressor scales; and whether or not the individual lost a loved one from COVID-19 (Table 3). Demographic variables such as gender, sexual minority status, and race/ethnicity were included as control variables. Results indicated that parental educational attainment (one parent having at least a bachelor’s degree) (OR = 0.88, 95% CI: 0.83, 0.92), psychological well-being (Diener scale) (OR = 0.39, 95% CI: 0.38, 0.41), and resilience (OR = 0.84, 95% CI: 0.82, 0.86) were protective factors for severe psychological distress. Risk factors for severe psychological distress included loneliness (OR = 1.82, 95% CI: 1.76, 1.85), COVID-19 related stress (OR = 1.61, 95% CI: 1.57, 1.66), and having lost a loved one from COVID-19 (OR = 1.34, 95% CI: 1.26, 1.42).

**Table 3:** Multiple logistic regression analysis of severe psychological distress.

	*B*	S.E.	Wald *χ*^2^	OR	95% CI	*p* value
Year in school	−0.006	0.010	0.37	0.99	0.97, 1.01	0.557
Parent bachelors	−0.132	0.025	26.85	0.88	0.83, 0.92	<0.001
Diener scale	−0.933	0.016	3518.25	0.39	0.38, 0.41	<0.001
Resilience scale	−0.177	0.014	168.66	0.84	0.82, 0.86	<0.001
Loneliness scale	0.591	0.014	1865.86	1.82	1.76, 1.85	<0.001
COVID stressors scale	0.478	0.013	1347.80	1.61	1.57, 1.66	<0.001
Lost a loved one from COVID	0.290	0.032	82.87	1.34	1.26, 1.42	<0.001
Interaction: non-binary by sexual minority	0.602	0.174	11.93	1.83	1.30, 2.57	<0.001

**Figure 2:**
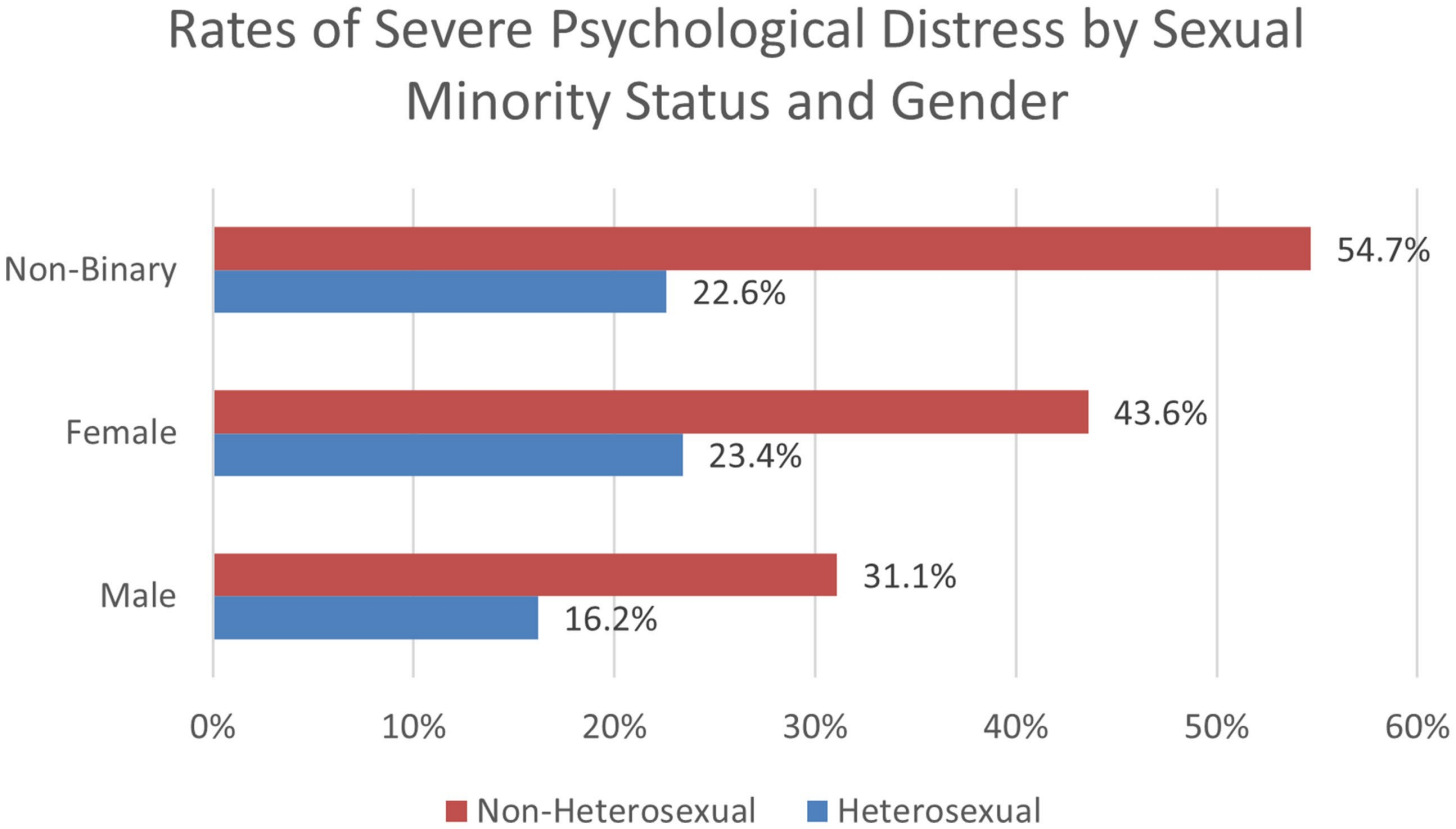
Rates of severe psychological distress by sexual minority status and gender (Spring 2021).

We conducted a logistic regression model with a series of two-way interaction terms for the gender, race/ethnicity, and sexual orientation variables. When analyzing gender and sexual minority status (Figure 2), a significant interaction was present for non-binary sexual minority students (OR = 1.83, 95% CI: 1.30, 2.57, *p* < 0.001). No significant interactions were found between gender and race/ethnicity, or for race/ethnicity and sexual minority status. The two-way interactions were significant when included in separate logistic regression models and when all three were included in a single model. A three-way interaction between gender, race, and sexual minority status was not found significant. These findings indicate that non-binary sexual minority students reported increased odds of severe psychological distress. These interaction findings indicate that students who were both non-binary and a sexual minority student were at an increased odds of experiencing severe psychological distress compared to the individual variables alone.

#### 3.2.1 Intersectionality for mental health symptoms

To explore the significant two-way interactions for mental health symptoms, we used chi-square analyses to examine proportions of the sample to investigate psychological distress by demographic categories. For White males, 16.6% reported severe psychological distress, compared to 25.8% of females and 48.9% of non-binary students. For non-White students, 20.4% of males, 31.5% of females, and 51.7% of non-binary students reported severe psychological distress. Regarding heterosexual students, 16.2% of males, 23.4% of females, and 22.6% of non-binary students reported severe psychological distress. For non-heterosexual students, 31.1% of males, 43.6% of females, and 54.7% of non-binary students reported severe psychological distress. When analyzing race and sexual minority status, we found that 18.4% of White and 24% of non-White heterosexual students reported severe psychological distress, compared to 41.5% of White and 45.5% of non-White sexual minority students. These findings are important to help identify how certain combinations of demographic subgroups may be at higher risk for severe psychological distress. Findings revealed that students who are both non-White and non-heterosexual experienced the greatest rates of psychological distress.

**Figure 3:**
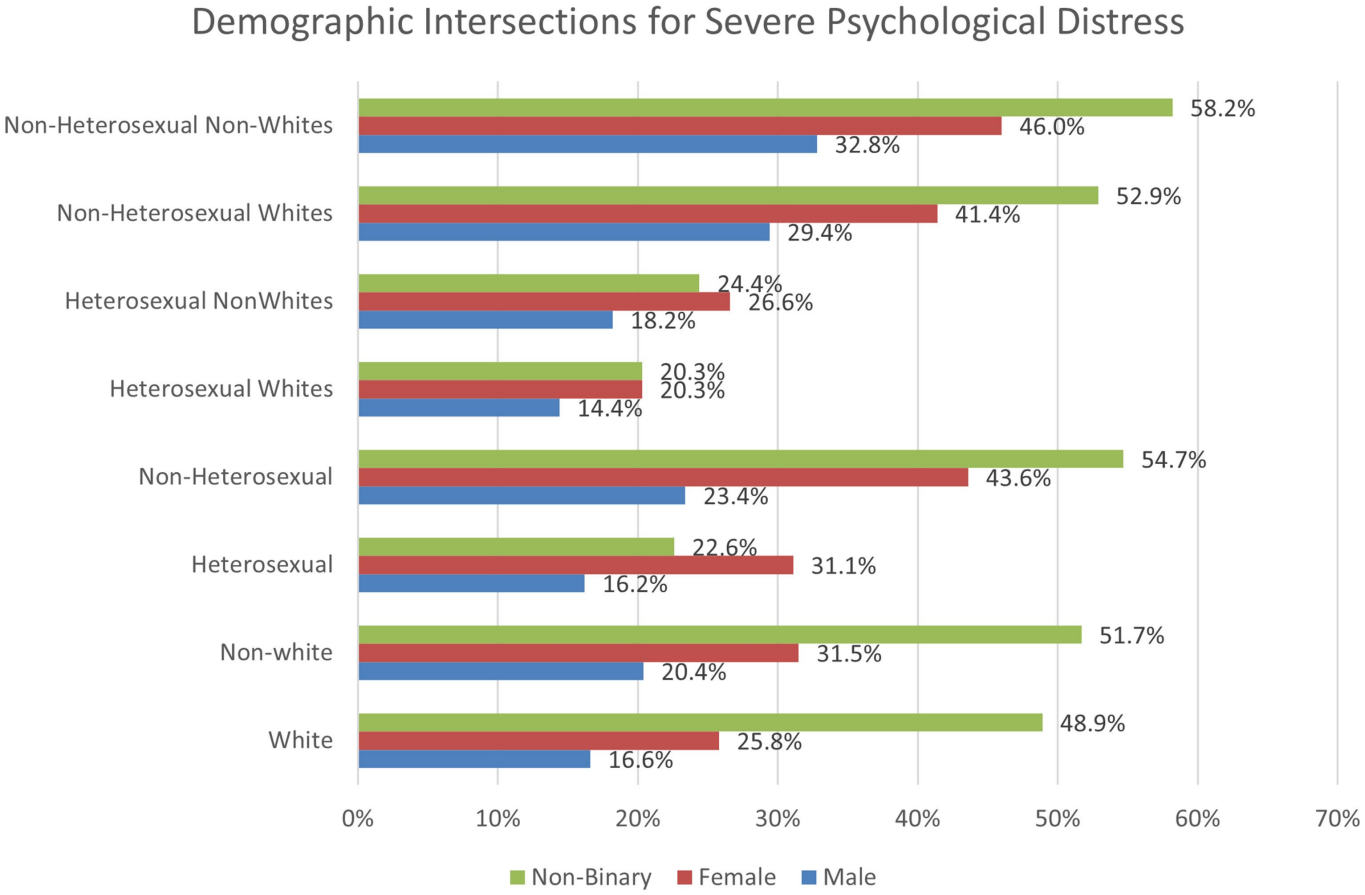
Two and three-way intersections between race/ethnicity, sexual minority status, and gender regarding severe psychological distress.

Finally, we conducted a chi-square test analyzing a three-way intersection between race/ethnicity, sexual minority status, and gender (Figure 3). For heterosexual Whites, 14.4% of males and 20.3% of both females and non-binary students reported severe psychological distress. For heterosexual non-Whites, 18.2% of males, 26.6% of females, and 24.4% of non-binary students suffered from severe psychological distress. For non-heterosexual White students, 29.4% of males, 41.4% of females, and 52.9% of non-binary students had high psychological distress. Non-heterosexual non-White students experienced the highest distress rates of 32.8% for males, 46% for females, and 58.2% for non-binary students. These findings are important to demonstrate how those of multiple minority identities are at greater risk of severe psychological distress. Findings indicated that students who identify as non-White, non-heterosexual, and non-binary experienced worsened psychological distress compared to any other combination of demographic groups (Figure 3).

### 3.3 Utilization of mental health services

Table 4 highlights Spring 2021 demographic variables for past year utilization of mental health services. Chi-square analyses indicated that for gender, males had the lowest percentage of students utilizing mental health services in the past year (17.1%), followed by females (30.2%), and more than half of the students who identify as non-binary accessed mental health services in the past year (54.6%). Regarding sexual orientation, percentages of non-heterosexual (45.6%) students who utilized past year services more than doubled the percentage of heterosexual students (21.7%). When looking at race and ethnicity data, despite Whites having the lowest reported severe psychological distress (K6 score), Whites were more likely to utilize mental health services in the past year (32.8%) compared to non-Whites (21.8%).

**Table 4:** Past year utilization of mental health services by demographic subgroup.

During pandemic: Spring 2021				
Demographic	Sub-group	*N*	Utilization %	Statistical significance
Gender	Male	15,529	17.1%	*F*(2, 56,792) = 989.09, *p* < 0.001
	Female	38,700	30.2%	
	Non-binary	2,566	54.6%	
Sexual orientation	Heterosexual	42,343	21.7%	*F*(1, 56,964) = 3279.30, *p* < 0.001
	Non-Heterosexual	14,623	45.6%	
Year in school	1st year	15,493	24.2%	*F*(4, 57,144) = 40.08, *p* < 0.001
	2nd year	13,166	27.9%	
	3rd year	14,942	29.1%	
	4th year	11,157	30.4%	
	5th year	2,391	30.0%	
Race/ethnicity	White	29,813	32.8%	*F*(5, 55,571) = 228.44, *p* < 0.001
	Asian	8,712	17.4%	
	Black or African American	1,807	25.8%	
	Hispanic	8,428	20.0%	
	Biracial or Multiracial	5,881	30.0%	
	Other	936	23.8%	
	Non-White total	25,764	21.9%	

We conducted a series of logistic regression analyses for past year mental health services utilization (Table 5) that included the following predictors: year in school, parent bachelor’s degree, severe psychological distress, resilience, loneliness, and COVID-19 stressor scales, and whether or not the individual lost a loved one from COVID-19. Demographic variables such as gender, sexual minority status, and race/ethnicity were included as control variables. Students who have at least one parent with a bachelor’s degree were more likely to access services (OR = 1.42, 95% CI: 1.36, 1.49), as well as those who suffered from loneliness (OR = 1.17, 95% CI: 1.14, 1.19) and stressors related to COVID-19 (OR = 1.05, 95% CI: 1.03, 1.07). Students with high resilience were less likely to utilize mental health services in the past year (OR = 0.85, 95% CI: 0.83, 0.87). Losing a loved one from COVID-19 was not found to be statistically significant for past year utilization of mental health services.

**Table 5:** Multiple logistic regression analysis of past year utilization of mental health services.

	*B*	S.E.	Wald χ^2^	OR	95% CI	*p* value
Health insurance	0.482	0.079	36.77	1.62	1.39, 1.89	<0.001
Year in school	0.117	0.009	177.88	1.12	1.11, 1.14	<0.001
Parent bachelors	0.351	0.023	233.07	1.42	1.36, 1.49	<0.001
Kessler Severe	0.528	0.025	431.40	1.70	1.61, 1.78	<0.001
Resilience scale	−0.165	0.011	222.02	0.85	0.83, 0.87	<0.001
Loneliness scale	0.155	0.012	176.68	1.17	1.14, 1.19	<0.001
COVID stressors scale	0.049	0.011	19.06	1.05	1.03, 1.07	<0.001
Lost a loved one from COVID	−0.025	0.030	0.71	0.98	0.92, 1.03	=0.400
Interaction: female by non-White	−0.246	0.054	20.87	0.78	0.70, 0.87	<0.001
Interaction: female by sexual minority	−0.133	0.061	4.77	0.88	0.78, 0.99	=0.029
Interaction: non-binary by sexual minority	0.389	0.147	6.97	1.48	1.11, 1.97	=0.008

**Figure 4:**
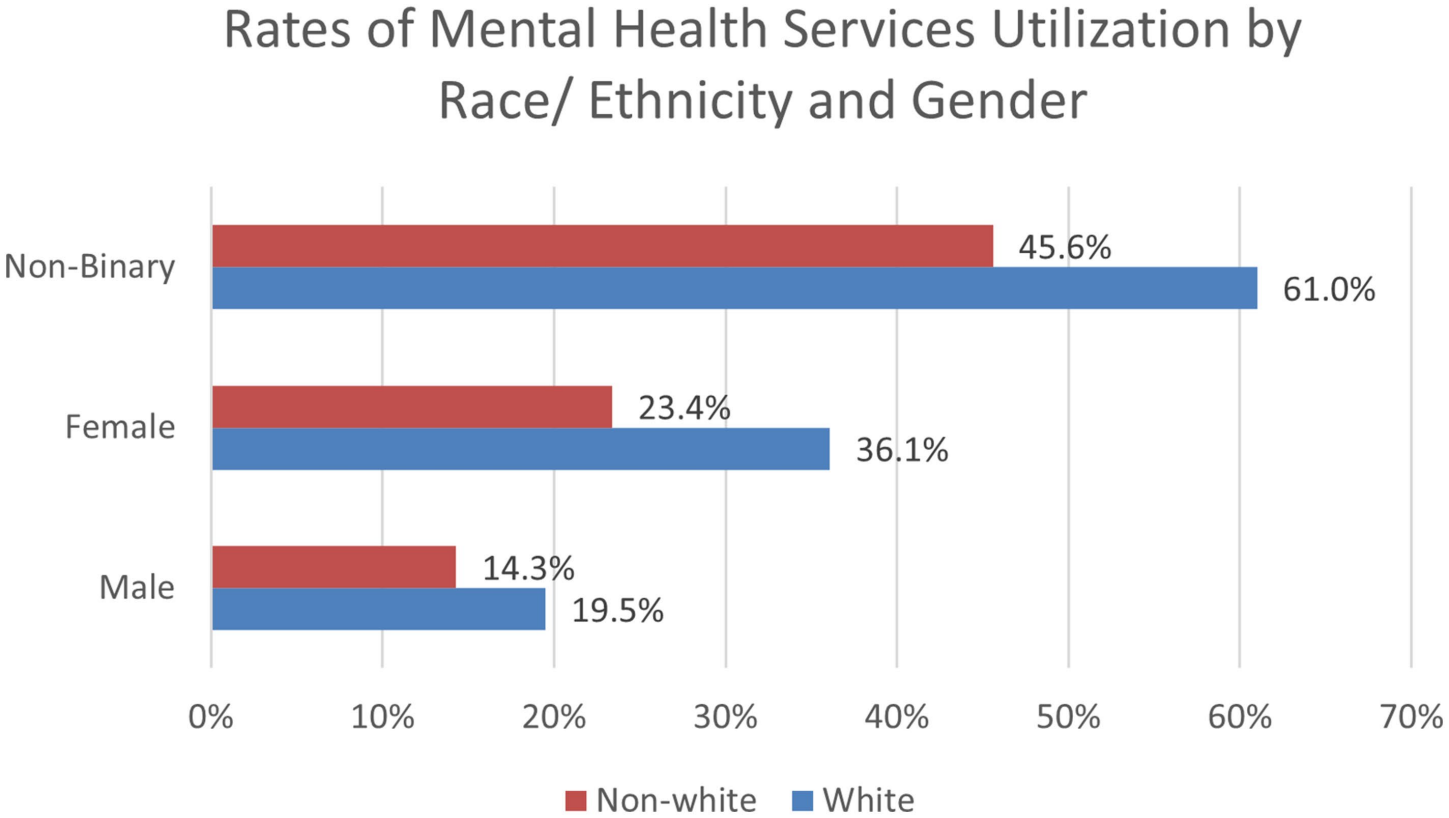
Rates of mental health services utilization by race/ethnicity and gender (Spring 2021).

**Figure 5:**
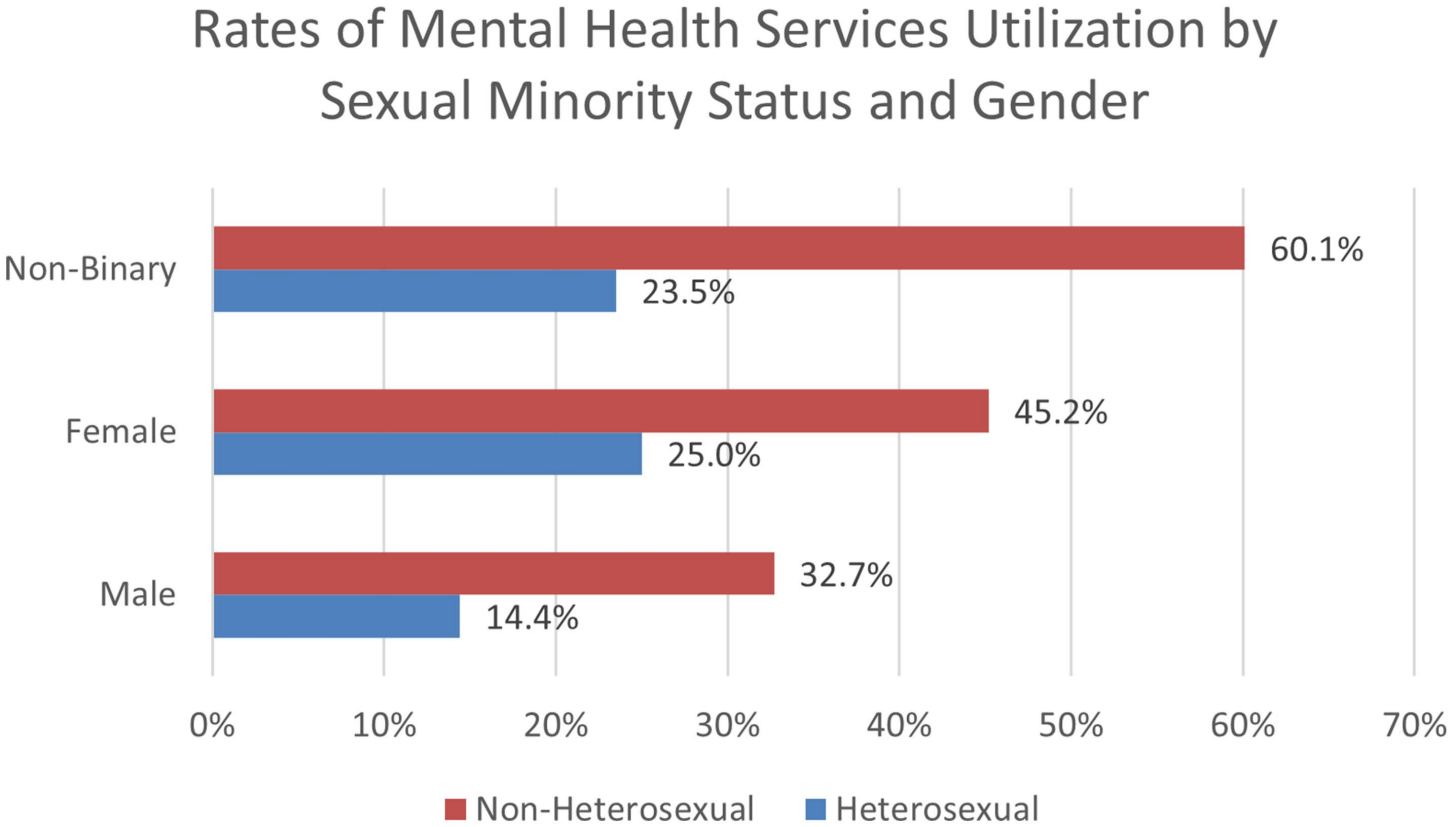
Rates of mental health services utilization by sexual minority status and gender (Spring 2021).

When exploring two-way interactions for past year utilization of mental health services, a positive interaction was found for non-White females (OR = 0.78, 95% CI: 0.70, 0.87, *p* < 0.001), which can be found in Figure 4. A positive interaction was also found for gender and sexual minority status (Figure 5). Compared to males, non-binary sexual minority students were most likely to access mental health services in the past year (OR = 1.48, 95% CI: 1.11, 1.97, *p* = 0.008). No significant interactions were found between race/ethnicity and sexual minority status for past year use of mental health services. The two-way interactions were significant when ran in separate logistic regression models and when all three combined into one model. A three-way interaction between gender, race, and sexual minority status was not found significant. These interaction findings indicate that students who are both non-White and female are at a decreased odds of accessing mental health services compared to these individual variables alone. These findings also indicate individuals who identify as both non-binary and as a sexual minority student are most likely to access mental health services.

#### 3.3.1 Intersectionality for utilization of mental health services

To explore the significant two-way interactions for utilization of mental health services, we used chi-square analyses to examine proportions of the sample by demographic categories. For White males, 19.5% utilized mental health services in the past year, compared to 36.1% of females and 61% of non-binary students. For non-White students, 14.3% of males, 23.4% of females, and 45.6% of non-binary students accessed services. For heterosexual students, 14.4% of males, 25% of females, and 23.5% of non-binary students received past year mental health services. For non-heterosexual students, 32.7% of males, 45.2% of females, and 60.1% of non-binary students utilized services. When analyzing race and sexual minority status, we found that 25.8% of White and 16.9% of non-White heterosexual students obtained mental health services, compared to 52.4% of White and 37.3% of non-White sexual minority students. These findings are important to help identify how certain demographic subgroups may be at a greater risk of not utilizing mental health services.

**Figure 6:**
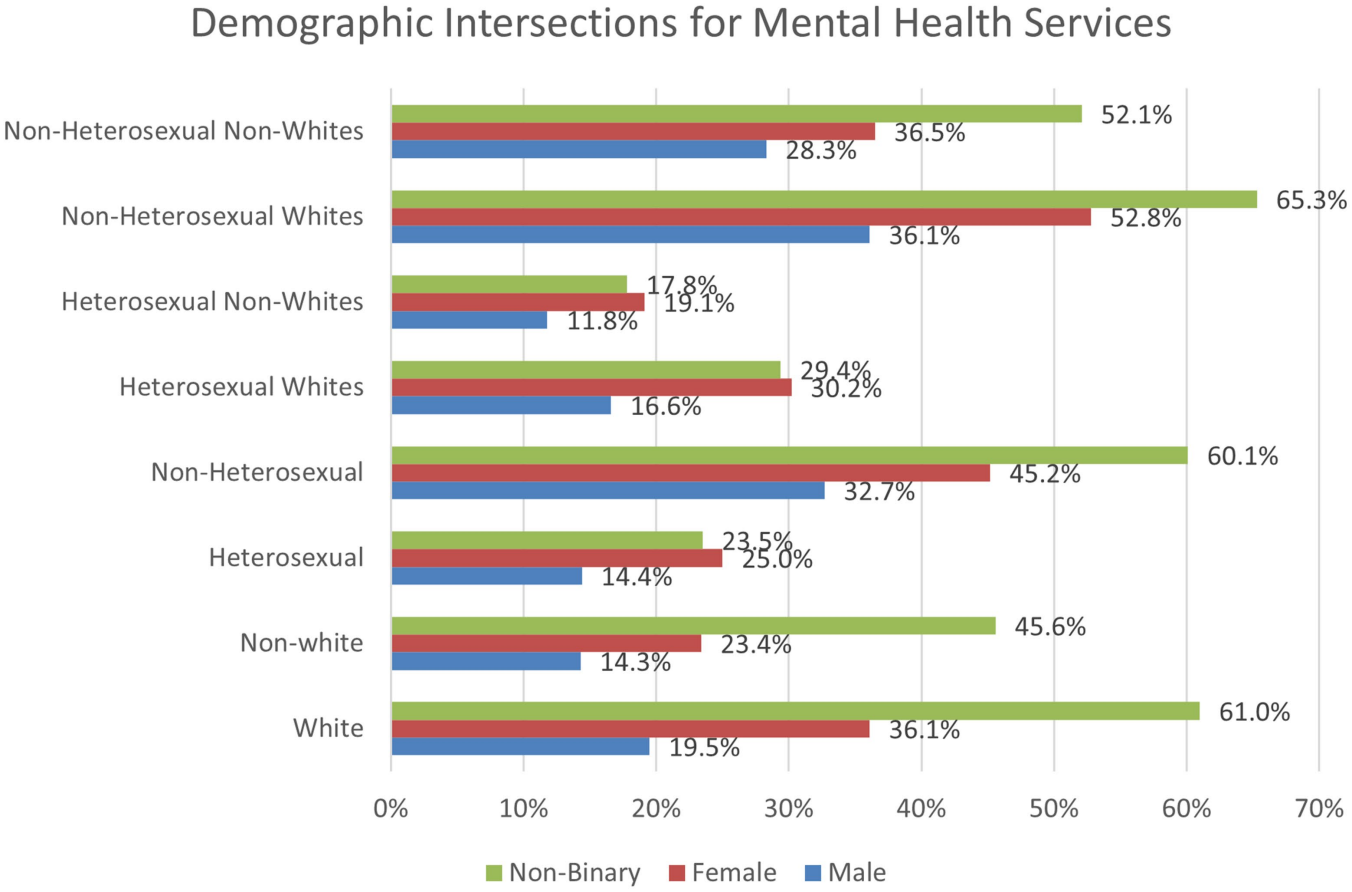
Two and three-way intersections between race/ethnicity, sexual minority status, and gender regarding past year access to mental health services.

Next, we conducted chi-square tests analyzing a three-way intersection between race/ethnicity, sexual minority status, and gender (Figure 6). For heterosexual Whites, 16.6% of males, 30.2% of females, and 29.4% of non-binary students utilized past year mental health services. For heterosexual non-Whites, these percentages dropped to 11.8% for males, 19.1% for females, and 17.8% for non-binary students. For non-heterosexual White students, 36.1% of males, 52.8% of females, and 65.3% of non-binary students reported seeking mental health services in the past year. These rates declined for non-heterosexual non-White students as 28.3% of males, 36.5% of females, and 52.1% of non-binary students utilized services.

## 4 Discussion

Analyses from this study revealed that several demographic groups were at an elevated risk for severe psychological distress during the peak of the pandemic, including females and sexual minority students. Consistent with intersectionality and minority stress theory, those who belong to more marginalized groups experienced worsened mental health outcomes. For instance, non-White students who identify as non-binary and non-heterosexual experienced the greatest rates of severe psychological distress. This is an important finding as recognition of intersectionality can help with health equity efforts. Parental education, psychological well-being, and resilience were all protective factors against psychological distress. Risk factors included loneliness, COVID-19 stressors, and having lost a loved one from COVID. These findings indicate groups who were at the greatest risk of poor mental health during the pandemic, along with factors that helped diminish these outcomes.

When analyzing past year utilization of mental health services during the peak of the pandemic, students of color were less likely than their White counterparts to utilize services, regardless of gender. Non-White female students were at a decreased odds of accessing mental health services, while non-binary sexual minority students utilized services more frequently. Students with parents who have a bachelor’s degree or higher, have health insurance, and reported loneliness were most likely to receive mental health services in the past year. Students who reported psychological well-being and resilience were least likely to utilize mental health services in the past year. These findings are important to better understand sub-groups who disproportionally suffer from severe psychological distress but may not be accessing the necessary care. University and community outreach efforts to non-White female college students may be important to help to increase utilization of mental health services for this demographic group.

Findings from this study are consistent with prior research related to diverse populations. Racial/ ethnic, gender, and sexual minority groups are typically at a greater risk of suffering from poor mental health outcomes due to factors such as cultural stigma, lack of access to high quality mental health services, discrimination, and general lack of awareness about mental health (25). Prior studies have also found that students of color utilize mental health services significantly less than White students (26). Additionally, those with multiple disadvantaged statuses have been found to often experience worse health outcomes compared to those who suffer from a single disadvantaged status (27).

It is important to better understand barriers to utilizing mental health services, especially among minority groups. A systematic review conducted by Lu et al. (28) analyzed barriers and facilitators related to utilization of mental health services among racial and ethnic minorities, and found that factors such as cultural stigma, low household income, and lack of health insurance decreased adolescents’ likelihood of utilizing adequate services. When developing innovative approaches to improving mental health outcomes on college campuses, it is important to be culturally sensitive and understand the diverse needs of the specific student population. A mixed-methods study completed during the pandemic revealed that students’ key priorities for quality well-being resources included that they must be easy to access, they must be inclusive and focus on prevention, and they must feel like a safe space (21). Improvements in mental health resources must also address stigma and empower students to access necessary care, such as use of peer support programs and normalizing mental health treatment.

Prior research has found that loneliness can induce or worsen instances of anxiety, depression, stress, and general mental health in college students (29). Previous studies have also found associations between low parental education and the onset of mental disorders in adolescence and adulthood (30). Parental education may be a pathway toward resources to promote recovery from mental health, such as access to health insurance and mental health treatment. Additionally, greater educational attainment is associated with reduced stigma toward mental illness (31), which could promote support toward mental health treatment.

The present study has several strengths and limitations. One strength includes the large sample size of college students geographically dispersed across the United States, which allowed the ability to assess intersectionality of how demographic variables are associated with psychological distress and utilization of mental health services. Additionally, this study included a wide variety of relevant risk and protective factors for psychological distress and mental health utilization, all of which used validated psychosocial measures with good psychometric properties.

Due to the cross-sectional nature of the study design, a limitation of this study is the inability to follow students over time to track long-term trends and causal relationships between COVID-19 stressors and mental health outcomes. Future studies should include longitudinal data to allow for a deeper understanding of how mental health trajectories evolve over time and how interventions might be tailored to different stages of distress.

Another limitation is the lack of information related to students’ use of telehealth for mental health services. As time went on during the pandemic, there was a large rise in virtual health services, including mental health treatment. Although telehealth is not a new concept, less than 1% of patients used these services prior to the pandemic for both mental health services and general outpatient doctor visits (visits other than mental health appointments) (32). This rate increased to 11% utilization for general outpatient visits, and 40% for mental health and substance use disorder visits toward the peak of the pandemic between March and August 2020 (32). While telehealth visits for general outpatient care steadily decreased once in-person services resumed, telehealth visits for mental health services have remained constant (32). This indicates that the rise of telehealth services played an important role in helping meet the rising needs of mental health services during the pandemic. A study conducted by Bulkes et al. (33) compared clinical outcomes of 1,192 United States adults who received in-person mental health treatment prior to the pandemic compared to a matched sample of 1,192 United States adults who received telehealth services during the pandemic. The study found no significant differences in mental health symptoms upon discharge, indicating that telehealth services can be a viable alternative to in-person mental health services (33). More research is needed to assess the effectiveness of telehealth services within university students, and future surveys should consider assessing student satisfaction with telehealth services and how they may impact barriers to receiving mental health services, such as accessibility.

The purpose of this study was to analyze changes in trends of mental health symptoms and utilization of mental health services in United States college students from before the COVID-19 pandemic to its’ peak, along with identifying high-risk groups and gaps in treatment. This research study is important in helping to identify students who were at the greatest risk of psychological distress and least likely to access necessary mental health services. The findings from this study are important to the field of public health so that tailored interventions can be developed to meet the needs of high-risk groups.

## 5 Conclusion

Findings from this study indicate that while United States college students experienced an increase in symptoms related to severe psychological distress and reductions in well-being from pre-COVID-19 to peak COVID-19, rates of mental health service utilization decreased during this same timeframe. This is an important gap because more United States college students are suffering from mental health concerns, but less are receiving necessary treatment. Intersectionality among multiple minoritized groups showed that the highest rates of psychological distress were found in non-binary sexual minority students of color, along with females. The largest gaps in utilization of mental health services were found in non-White students, especially female students of color. Future research is needed to increase our understanding of the barriers to mental health service use among high-risk university students.

## Data Availability

Publicly available datasets were analyzed in this study. This data can be found here: American College Health Association (https://www.acha.org/ncha/).

## References

[1] Chen JA Stevens C Wong SH Liu CH. Psychiatric symptoms and diagnoses among US college students: a comparison by race and ethnicity. Psychiatr Serv. (2019) 70:442–9. doi: 10.1176/appi.ps.201800388, PMID: 30914001 PMC6628693

[2] Roche AI Holdefer PJ Thomas EB. College student mental health: understanding changes in psychological symptoms in the context of the COVID-19 pandemic in the United States. Curr Psychol. (2022) 43:1–10. doi: 10.1007/s12144-022-03193-w 35645550 PMC9124747

[3] World Health Organization (WHO) (2022). The impact of COVID-19 on mental health cannot be made light of. Available online at: https://www.who.int/news-room/feature-stories/detail/the-impact-of-covid-19-on-mental-health-cannot-be-made-light-of (Accessed March 27, 2023).

[4] Abrams Z. Student mental health in crisis. Campuses are rethinking their approach. Monit Psychol. (2022) 53:60.

[5] Frazier P Liu Y Asplund A Meredith L Nguyen-Feng VN. US college student mental health and COVID-19: comparing pre-pandemic and pandemic timepoints. J Am Coll Heal. (2021) 71:1–11. doi: 10.1080/07448481.2021.1987247 34762560

[6] World Health Organization (WHO) (2021). Mental health of adolescents. Available online at: https://www.who.int/news-room/fact-sheets/detail/adolescent-mental-health (Accessed March 9, 2023).

[7] Moore SE Wierenga KL Prince DM Gillani B Mintz LJ. Disproportionate impact of the COVID-19 pandemic on perceived social support, mental health and somatic symptoms in sexual and gender minority populations. J Homosex. (2021) 68:577–91. doi: 10.1080/00918369.2020.1868184, PMID: 33399504

[8] Barbayannis G Bandari M Zheng X Baquerizo H Pecor K Ming X. Academic stress and mental well-being in college students: correlations, affected groups, and COVID-19. Front Psychol. (2022) 13:886344. doi: 10.3389/fpsyg.2022.886344 35677139 PMC9169886

[9] Samek DR Akua BA Crumly B Duke-Marks A. Increasing mental health issues in college students from 2016-2019: assessing the intersections of race/ethnicity, gender, and sexual orientation. J Affect Disord. (2024) 354:216–23. doi: 10.1016/j.jad.2024.03.068, PMID: 38484884

[10] Zimmermann M Bledsoe C Papa A. Initial impact of the COVID-19 pandemic on college student mental health: a longitudinal examination of risk and protective factors. Psychiatry Res. (2021) 305:114254. doi: 10.1016/j.psychres.2021.114254, PMID: 34763271 PMC8556872

[11] Hall SS Zygmunt E. “I hate it here”: mental health changes of college students living with parents during the COVID-19 quarantine. Emerg Adulthood. (2021) 9:449–61. doi: 10.1177/21676968211000494

[12] Kim H Rackoff GN Fitzsimmons-Craft EE Shin KE Zainal NH Schwob JT. College mental health before and during the COVID-19 pandemic: results from a nationwide survey. Cogn Ther Res. (2022) 46:1–10. doi: 10.1007/s10608-021-10241-5, PMID: 34177004 PMC8214371

[13] Lee J Jeong HJ Kim S. Stress, anxiety, and depression among undergraduate students during the COVID-19 pandemic and their use of mental health services. Innov High Educ. (2021) 46:519–38. doi: 10.1007/s10755-021-09552-y, PMID: 33907351 PMC8062254

[14] Garcia Colato E Ludema C Rosenberg M Kianersi S Luetke M Chen C. The association between social factors and COVID-19 protective behaviors and depression and stress among midwestern US college students. PLoS One. (2022) 17:e0279340. doi: 10.1371/journal.pone.0279340, PMID: 36534666 PMC9762587

[15] Assari S. Parental educational attainment and mental well-being of college students: diminished returns of blacks. Brain Sci. (2018) 8:193. doi: 10.3390/brainsci8110193, PMID: 30380617 PMC6266217

[16] Zhao S Yiyue G. The effects of mother's education on college student's depression level: the role of family function. Psychiatry Res. (2018) 269:108–14. doi: 10.1016/j.psychres.2018.08.030, PMID: 30145289

[17] Lund IO Andersen N Handal M Ask H Skurtveit S Ystrom E. Parental drinking, mental health and education, and extent of offspring’s healthcare utilisation for anxiety/depression: a HUNT survey and registry study. Scand J Public Health. (2023) 51:902–10. doi: 10.1177/14034948221076212, PMID: 35331062

[18] Bauer GR Churchill SM Mahendran M Walwyn C Lizotte D Villa-Rueda AA. Intersectionality in quantitative research: a systematic review of its emergence and applications of theory and methods. SSM Popul Health. (2021) 14:100798. doi: 10.1016/j.ssmph.2021.100798, PMID: 33997247 PMC8095182

[19] Frost DM Meyer IH. Minority stress theory: application, critique, and continued relevance. Curr Opin Psychol. (2023) 51:101579. doi: 10.1016/j.copsyc.2023.101579, PMID: 37270877 PMC10712335

[20] American Psychological Association (2018). APA Dictionary of Psychology. Available online at: https://dictionary.apa.org/psychological-distress (Accessed August 5, 2024).

[21] Remskar M Atkinson MJ Marks E Ainsworth B. Understanding university student priorities for mental health and well-being support: a mixed-methods exploration using the person-based approach. Stress Health. (2022) 38:776–89. doi: 10.1002/smi.3133, PMID: 35137525 PMC9790713

[22] Goodwin J Behan L Kelly P McCarthy K Horgan A. Help-seeking behaviors and mental well-being of first year undergraduate university students. Psychiatry Res. (2016) 246:129–35. doi: 10.1016/j.psychres.2016.09.015, PMID: 27693865

[23] American Psychiatric Association (2023a). Mental Health Disparities: Diverse Populations. Available online at: https://www.psychiatry.org/psychiatrists/diversity/education/mental-health-facts#:~:text=Racial%2Fethnic%2C%20gender%2C%20and,of%20awareness%20about%20mental%20health (Accessed February 12, 2023).

[24] National Institute of Mental Health (NIMH) (2023). Mental Illness. Available online at: https://www.nimh.nih.gov/health/statistics/mental-illness#:~:text=Prevalence%20of%20Any%20Mental%20Illness%20(AMI),-Figure%201%20shows&text=This%20number%20represented%2022.8%25%20of,50%20and%20older%20(15.0%25) (Accessed March 27, 2023).

[25] American Psychiatric Association (2023b). Stigma, Prejudice, and Discrimination Against People with Mental Illness. Available online at: https://www.psychiatry.org/patients-families/stigma-and-discrimination (Accessed February 12, 2023).

[26] Lipson SK Zhou S Abelson S Heinze J Jirsa M Morigney J. Trends in college student mental health and help-seeking by race/ethnicity: findings from the national healthy minds study, 2013–2021. J Affect Disord. (2022) 306:138–47. doi: 10.1016/j.jad.2022.03.038, PMID: 35307411 PMC8995361

[27] Vohra-Gupta S Petruzzi L Jones C Cubbin C. An intersectional approach to understanding barriers to healthcare for women. J Community Health. (2023) 48:89–98. doi: 10.1007/s10900-022-01147-8, PMID: 36273069 PMC9589537

[28] Lu W Todhunter-Reid A Mitsdarffer ML Muñoz-Laboy M Yoon AS Xu L. Barriers and facilitators for mental health service use among racial/ethnic minority adolescents: a systematic review of literature. Front Public Health. (2021) 9:641605. doi: 10.3389/fpubh.2021.641605, PMID: 33763401 PMC7982679

[29] Campbell F Blank L Cantrell A Baxter S Blackmore C Dixon J. Factors that influence mental health of university and college students in the UK: a systematic review. BMC Public Health. (2022) 22:1778. doi: 10.1186/s12889-022-13943-x, PMID: 36123714 PMC9484851

[30] McLaughlin KA Breslau J Green JG Lakoma MD Sampson NA Zaslavsky AM. Childhood socio-economic status and the onset, persistence, and severity of DSM-IV mental disorders in a US national sample. Soc Sci Med. (2011) 73:1088–96. doi: 10.1016/j.socscimed.2011.06.011, PMID: 21820781 PMC3191493

[31] Corrigan PW Watson AC. The stigma of psychiatric disorders and the gender, ethnicity, and education of the perceiver. Community Ment Health J. (2007) 43:439–58. doi: 10.1007/s10597-007-9084-9 17876705

[32] Lo J. Rae M. Amin K. Cox C. Panchal N. Miller B. (2022). Telehealth has played an outsized role meeting mental health needs during the COVID-19 pandemic. Kaiser Family Foundation. Available online at: https://www.kff.org/coronavirus-covid-19/issue-brief/telehealth-has-played-an-outsized-role-meeting-mental-health-needs-during-the-covid-19-pandemic/ (Accessed March 1, 2023).

[33] Bulkes NZ Davis K Kay B Riemann BC. Comparing efficacy of telehealth to in-person mental health care in intensive-treatment-seeking adults. J Psychiatr Res. (2022) 145:347–52. doi: 10.1016/j.jpsychires.2021.11.003, PMID: 34799124 PMC8595951

